# Flavin Conjugated Polydopamine Nanoparticles Displaying Light-Driven Monooxygenase Activity

**DOI:** 10.3389/fchem.2019.00278

**Published:** 2019-04-26

**Authors:** Leander Crocker, Ljiljana Fruk

**Affiliations:** Department of Chemical Engineering and Biotechnology, University of Cambridge, Cambridge, United Kingdom

**Keywords:** flavin, polydopamine, light-driven reaction, monooxygenase, indole

## Abstract

A hybrid of flavin and polydopamine (PDA) has been explored as a photocatalyst, drawing inspiration from natural flavoenzymes. Light-driven monoxygenase activity has been demonstrated through the oxidation of indole under blue light irradiation in ambient conditions, to afford indigo and indirubin dyes. Compared to riboflavin, a flavin-polydopamine hybrid is shown to be more resistant to photobleaching and more selective toward dye production. In addition, it has been demonstrated that it can be recycled from the solution and used for up to four cycles without a marked loss of activity, which is a significant improvement compared to other heterogenous flavin catalysts. The mechanism of action has been explored, indicating that the PDA shell plays an important role in the stabilization of the intermediate flavin-peroxy species, an active component of the catalytic system rather than acting only as a passive nanocarrier of active centers.

## Introduction

Flavin-containing monooxygenases (FMOs) are an important class of xenobiotic-degrading enzymes present in both eukaryotic and prokaryotic organisms. For example, they are able to add molecular oxygen to the lipophilic xenobiotic compounds, thereby increasing their solubility enough to allow excretion. As a result, organisms are protected from potentially toxic exogenous compounds derived from natural sources and, particularly important for humans, the metabolism of drugs and pollutants (Krueger and Williams, 2005; Zhou and Shephard, 2006; Hodgson, 2010). One of the substrates for FMOs, which is also widespread in nature is the *N*-heterocycle indole. It is considered to be an aromatic pollutant due to its toxicity and potential mutagenicity (Sullivan and Gad, 2014), but it is also a versatile intermediate species and signaling molecule across families of organisms (Lee and Lee, 2010; Erb et al., 2015; Lee et al., 2015a,b). Indigo dyes are currently commercially produced by chemical synthesis using aniline, formaldehyde, and hydrogen cyanide to form phenylglycinonitrile, subsequently hydrolyzed to yield phenylglycine, which is finally converted to indigotin. This process involves use of toxic chemicals and extensive purification steps, limiting the environmental viability and prompting the design of more green-chemistry oriented strategies (Blackburn et al., 2009).

**Scheme 1:**
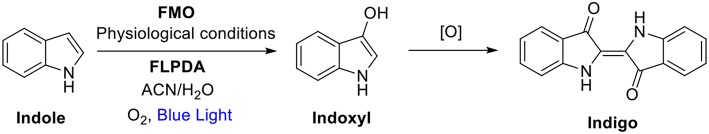
**Indole oxidation to indoxyl and further oxidation and dimerization to indigo where FMO, Flavin-containing monooxygenases; **FLPDA**, flavin-polydopamine; CAN, acetonitrile; [O], further oxidation processes by O_2_ (Mermod et al., 1986; Meyer et al., 2002)**.

FMOs, as well as the other xenobiotic-degrading enzymes such as cytochrome P450s, have been shown to convert indole to the blue indigo dye through initial oxidation to indoxyl and subsequent dimerization to form the dye as shown in Scheme 1. This transformation has been utilized as an enzymatic assay to screen for oxygenases (O'Connor et al., 1997; Singh et al., 2010; Lin et al., 2012; Nagayama et al., 2015), but also as a greener alternative to the industrial manufacturing of indigo and related indigoid dyes. To achieve that, a whole cell biocatalysis relying on the cellular FMO's is utilized to achieve that since the current industrial manufacturing of these dyes is highly energy demanding, results in large amounts of toxic waste products, and requires number of purification steps (Han et al., 2012; Hsu et al., 2018; Ma et al., 2018). However, a whole-cell biocatalysis is also faced with complex product separation and catalyst inhibition, which incurs large costs and reactor downtime periods (Lin and Tao, 2017). Alternatives such as the use of the isolated enzymes suffer from limited enzyme quantities, low stability under non-physiological conditions, sensitivity to organic solvents, as well as challenging post-reaction isolation, hence limiting large scale industrial applications (Reetz, 2002; Li et al., 2012).

Taken out of a protein environment, flavin analogs derived from cofactors flavin mononucleotide (FMN), flavin adenine dinucleotide (FAD) and riboflavin have shown huge potential for catalytic applications, especially in photocatalytic processes (de Gonzalo and Fraaije, 2013; König et al., 2013; Cibulka, 2015). Despite the relative ease of visible-light driven flavin photocatalysis, high yielding reactions, and low toxicity, there are still challenges related to their long-term stability and photobleaching, as well as the separation of the catalyst from the products and subsequent catalyst reuse. To address these issues, attempts have been made to immobilize flavins onto various solid carriers such as silica beads or resins to achieve a heterogeneous catalytic systems, although limited success has been reported in relation to recyclability and activity (Schmaderer et al., 2009; Špačková et al., 2017; Arakawa et al., 2018). Taking this into account, we have rationalized that the use of solid, polymeric carrier, which not only permits immobilization of flavin but also displays some intrinsic properties similar to protein shells such as H-bonding and electron transfer, could significantly improve both the activity and post-reaction recovery of the hybrid catalytic system.

Herein, we present a new strategy to design a versatile enzyme-inspired photocatalytic system by embedding flavin within an active polymer matrix. We have chosen polydopamine (PDA) as the carrier polymer due to its reported biocompatibility (Hong et al., 2011; D'Ischia et al., 2014) and structural and electronic properties (Liu et al., 2014). Composed of a sequence of extended π-systems, PDA is considered to be an amorphous organic semiconductor, and has been used to improve various nanoparticle catalysts (Ma et al., 2015; Kunfi et al., 2017; Zhou et al., 2017), enhance the efficiency and stability of whole cell biocatalytic systems (Wang et al., 2017), and act as an electron gate for artificial photosynthesis due to its excellent electron accepting ability (Kim et al., 2014). Although PDA's main application has been to act as a molecular adhesive, due to its capability to form coatings on virtually any surface (Lee et al., 2007), an ability equally as interesting is its intrinsic catalytic activity (Mrõwczynski et al., 2014; Yang et al., 2014; Du et al., 2015), which has not yet been explored to afford hybrid organic systems with photocatalytic activity.

**Scheme 2:**
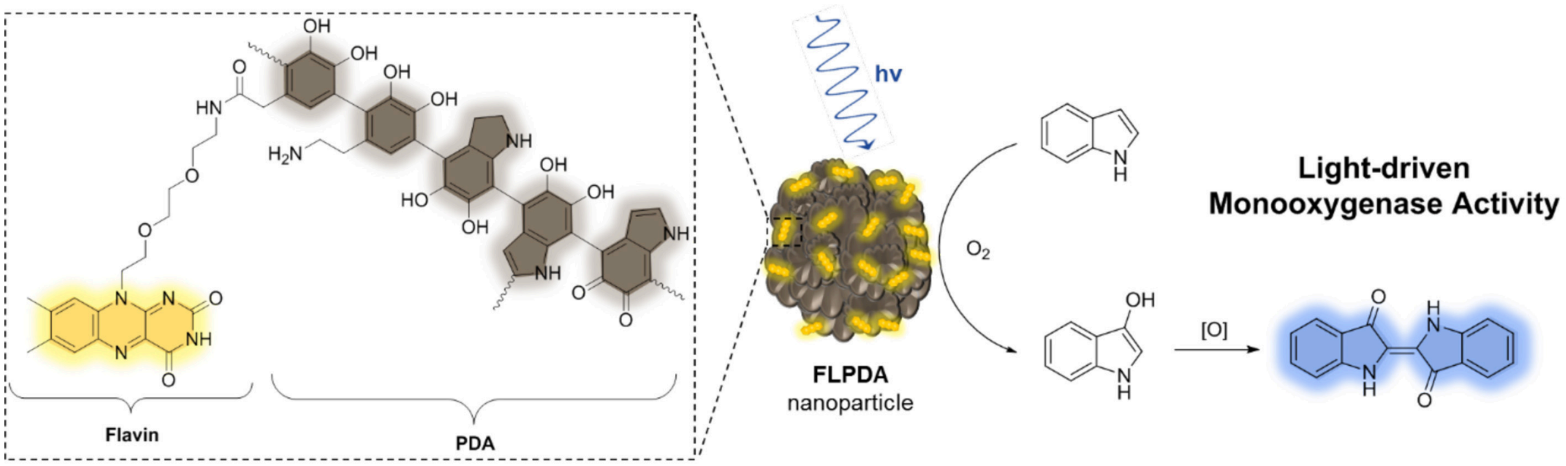
**Structure of flavin-polydopamine (FLPDA) units (left) and the reaction scheme illustrating explored light-driven oxidation of indole to indigo in presence of **FLPDA** nanoparticles (right)**.

We hypothesized that monoxygenase activity of the flavin-polydopamine (**FLPDA**) system could be initiated through blue light irradiation to excite the flavin moieties, bypassing the use of external reducing agents or cofactors such as NADH. At the same time, PDA can be utilized as an active solid support enabling both the electron transfer and the stabilization of reactive intermediates. The latter was inspired by BLUF (blue light sensors using FAD) photoreceptors, in which an electron transfer between flavin cofactor and tyrosine in the protein shell is the key process enabling the stabilization of intermediates and switching from dark-adapted to light-adapted state, which guides biological signaling pathways (Mathes et al., 2012). In addition, the photoinduced electron transfer from tryptophan to flavin has been shown to be a crucial step in the control of CRY protein's activity, which is responsible for control of the light dependent circadian clocks in plants and animals (Lin et al., 2018). As shown in Scheme 2, the structure of PDA mimics both the tyrosine and tryptophan residues, indicating that the polymer could actively engage in electron transfer processes and impact the catalytic activity of embedded flavin. With this in mind, we focused our efforts to synthesize **FLPDA** nanoparticles through the co-polymerization of a flavin-dopamine monomer, **FLDA**, and dopamine. We then investigated the light-driven monooxygenase activity of the particles through blue light irradiation in the presence of indole under ambient conditions.

## Experimental

### General

All materials were purchased from either Acros Organics, Alfa Aeser, Sigma-Aldrich, or TCI Chemicals in the highest purity available and used without further purification. ^1^H and ^13^C NMR measurements were carried out using a 500 MHz DCH Cryoprobe Spectrometer. HRMS was recorded on a ThermoFinnigan Orbitrap Classic (Fisher Scientific). UV-Vis absorption spectra were obtained with an Agilent Cary 300 Spectrophotometer. Fluorescence emission spectra were obtained using a Varian Cary Eclipse Fluorescence Spectrophotometer using excitation and emission slits of 10 nm. DLS and Zeta Potential measurements were recorded using a Zetasizer Nano Range instrument (Malvern Panalytical). FTIR spectroscopy was carried out using a Bruker Tensor 27 spectrometer with samples pressed into KBr pellets. STEM images were obtained using a Hitachi S-5500 In-Lens FE STEM (2009) at an acceleration voltage of 1.0 kV. Samples were suspended in water and drop cast on lacey carbon copper grids (Agar Scientific). HPLC was carried out on an Agilent 1260 Infinity Quaternary LC equipped with a Zorbax Eclipse Plus C18 column (5 μm, 4.6 × 1.5 mm, Agilent) and diode array detector (monitoring at 270 nm). The mobile phase consisted of Solvent A (water + 0.1% formic acid) and solvent B (ACN/MeOH, 50:50, v/v) running along the following gradient: 0–8 min 85% A and 15% B at a flow rate of 1 mL/min, 8–15 min 65% A and 35% B at a flow rate of 2 mL/min. Indole (99%), isatin (97%), and oxindole (98%) were purchased from Sigma-Aldrich and calibration curves were obtained from 0 to 1.0 mM stock solutions in order to estimate product concentrations and % conversions. LC-MS was performed on an Agilent G6550 QTof mass spectrometer coupled to an Agilent 1200 Series Infinity LC system using a Zorbax Eclipse Plus C18 column (5 μm, 4.6 × 1.5 mm, Agilent). The mobile phase consisted of Solvent A (water + 0.1% formic acid) and solvent B (ACN + 0.1% formic acid) running along the following gradient: 0–14 min 85% A and 15% B to 5% A and 95% B at a flow rate of 0.8 mL/min. The electrospray source was operated with a capillary voltage of 3.0 kV and a nozzle voltage of 1.0 kV. Nitrogen was used as the desolvation gas at a total flow of 14 L/min. All *m/z* values stated are that of the [M+H]^+^ molecular ion.

### Synthesis of FLDA Monomer

See section Synthesis of Flavin-Dopamine Monomer in Supplementary Information for full synthetic details.

### Synthesis of FLPDA

A mixture of ammonia solution (0.1 mL, 28%), ethanol (1.5 mL), and Milli Q water (4.5 mL) was stirred at room temperature for 30 min in reaction vessels protected from direct sunlight. Dopamine hydrochloride (15.80 mg, 0.083 mmol) dissolved in Milli-Q water (0.5 mL) and FLDA (8.73 mg, 0.017 mmol) dissolved in ethanol (0.5 mL) were mixed before being added dropwise to the reaction mixture. The resulting dark brown/black mixture was left to stir in the presence of air for 24 h. The mixture was then centrifuged at 25,000 × g for 30 min and the supernatant was removed. The precipitate was washed with Milli-Q water (3 × 40 mL) and then suspended in Milli-Q water (20 mL), frozen in liquid N_2_ and lyophilized to yield a dark brown/black powder (5.5 mg, ρ = 22% where ρ is the percent weight conversion of monomers) (Jiang et al., 2014).

### Photooxidation of Indole

**FLPDA** (0.01–0.1 mg/mL) and indole (0.2 μmol−0.05 mmol) were added to a 50:50 acetonitrile/water (2 mL) solvent system and the mixture was saturated with O_2_ gas for 10 min before irradiating with a custom-made blue LED strips setup (12 V) with a cooling fan to maintain a temperature of ~25°C (see Figure S1 for setup). For small scale reactions, 100 μL aliquots were taken from the reaction mixture and diluted to 1 mL (50:50 acetonitrile/water) for analysis by UV-Vis absorption spectroscopy. Post-irradiation (2–6 h), the reaction mixture was either diluted in 50:50 acetonitrile/water (10 mL) and the catalyst removed by centrifugation and washed with water (3 × 12 mL) for further use, or the catalyst was removed by centrifugation and the supernatant analyzed by HPLC-UV and LC-MS. For larger scale reactions the resulting supernatant was concentrated under reduced pressure and residue analyzed by ESI-MS and UV-Vis absorption spectroscopy.

## Results and Discussion

### Synthesis of Flavin-Polydopamine

**Scheme 3:**
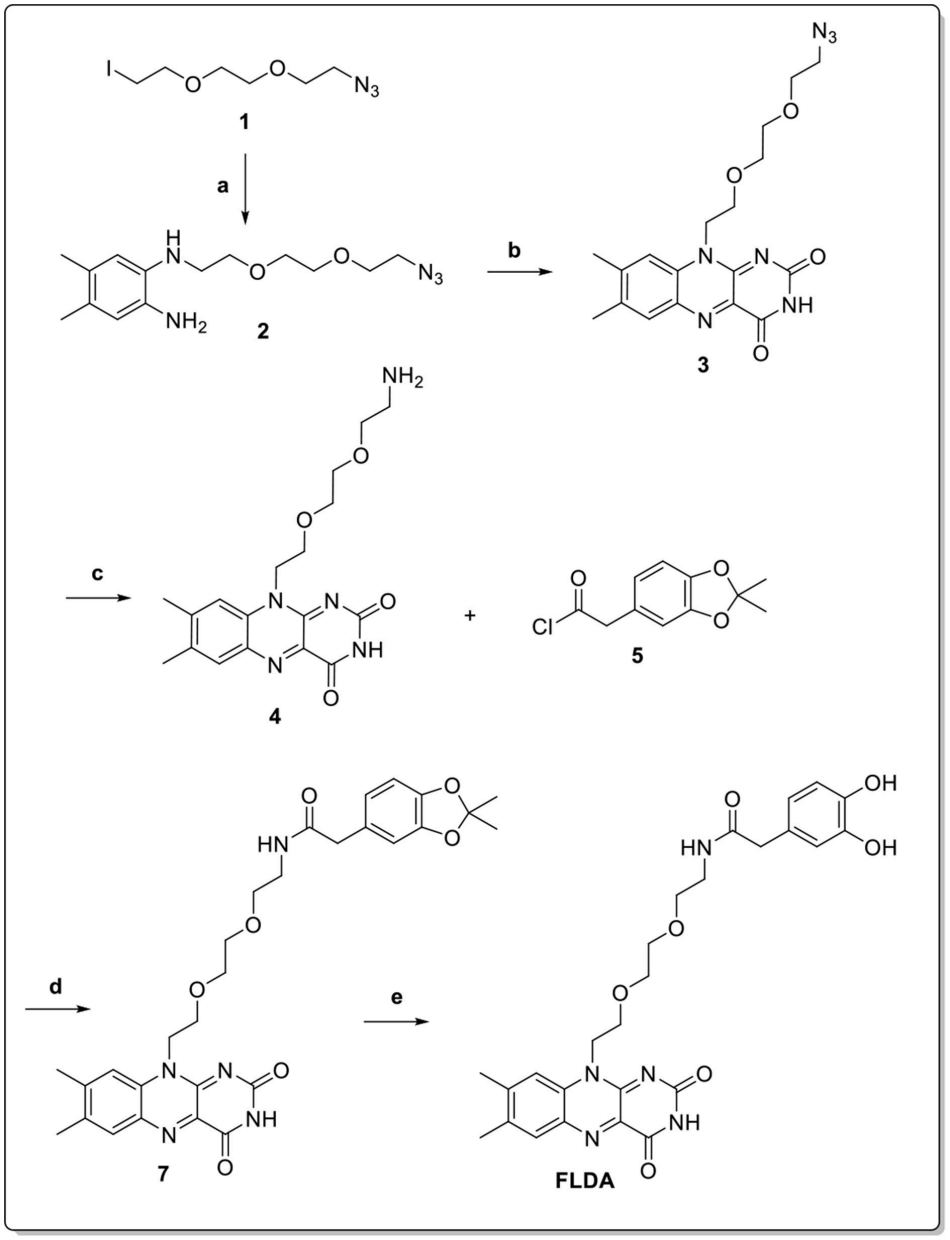
**Reaction conditions: (a) 4,5-dimethylbenzene-1,2-diamine, K_2_CO_3_, DMF, 50 °C, 12 h (70%); (b) alloxan monohydrate, B_2_O_3_, AcOH, RT, dark, 48 h (69%); (c) Pd/C, H_2_, AcOH, RT, dark, 18 h (84%); (d) TEA, DMF, RT, dark, 18 h (62%); (e) TFA/DCM, RT, dark, 2 h (99%)**.

**FLPDA** nanoparticles were formed by the copolymerization of dopamine (**DA**) with the flavin derivative, **FLDA** that was synthesized according to the route shown in Scheme 3. First, the functionalized triethylene glycol species, **1** (Deng et al., 2011) was used in a mono-substitution reaction with 4,5-dimethylbenzene-1,2-diamine to form *N*^1^-(2-(2-(2-azidoethoxy)ethoxy)ethyl)-4,5-dimethylbenzene-1,2-diamine, **2**.

Isoalloxazine formation was achieved through the double condensation reaction with alloxan monohydrate to obtain flavin derivative **3**. The azide functionality was then reduced *via* catalytic hydrogenation to yield the amine-bearing flavin derivative **4**. Conjugation of this compound to the activated catechol-protected dopamine analog **6** resulted in protected **FLDA** derivative **7**, which was subsequently deprotected to afford the target **FLDA** monomer. Co-polymerization of dopamine and **FLDA** was carried out following a room temperature procedure adapted from Ai et al. (2013), and using ammonia addition to a water/ethanol solvent system in the presence of air. It should be noted that the reaction vessels were protected from direct light exposure to avoid any possible side reactions through the excitation of flavin moieties.

**Figure 1:**
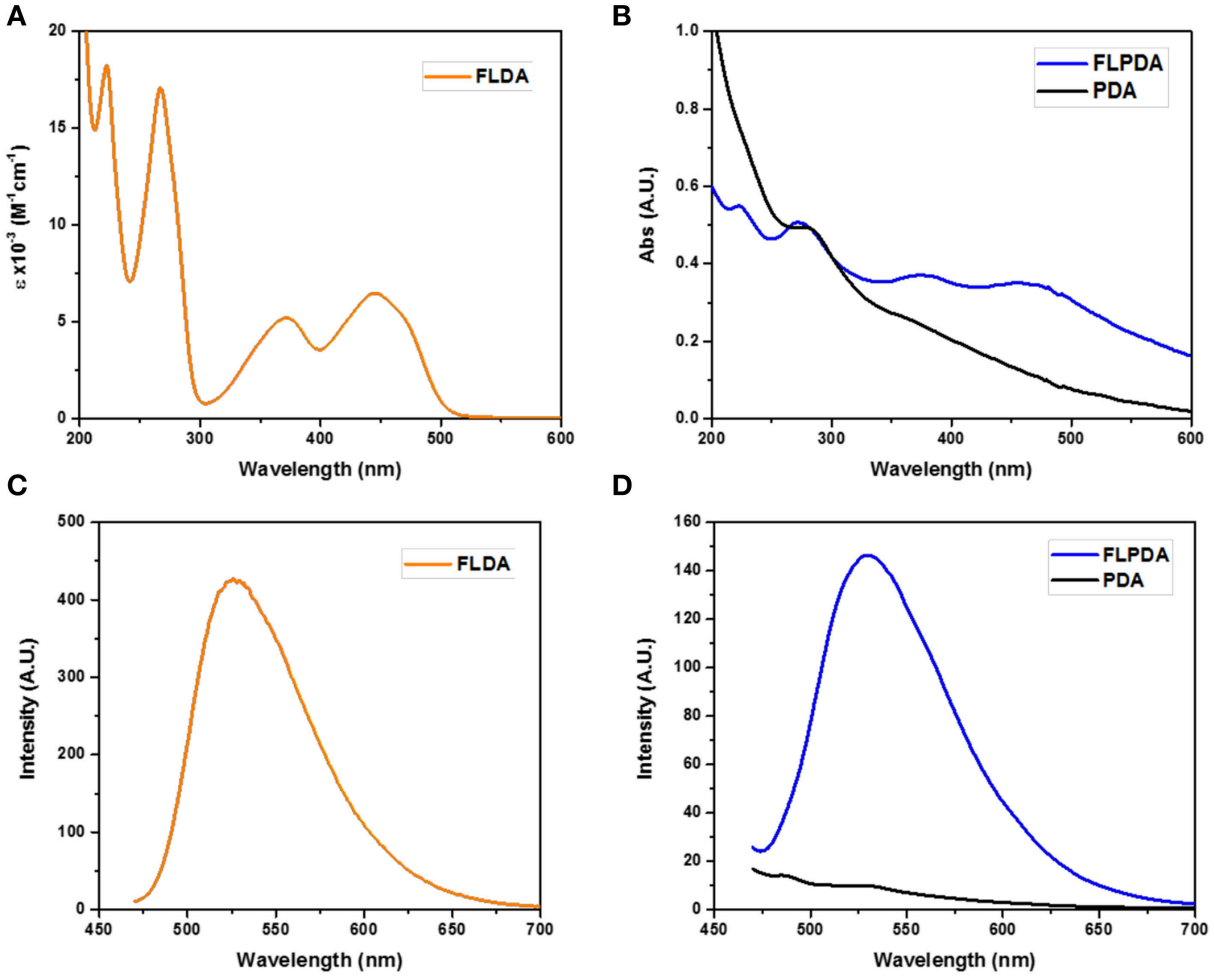
UV-Vis spectra of **FLDA** (80 μM, 0.04 mg/mL in water **(A)**, and **FLPDA** and PDA (0.1 mg/mL in water **(B)**, and corresponding fluorescence spectra **(C,D)**.

To validate the presence of flavin moieties, **FLPDA** was first analyzed by UV-Vis absorption and fluorescence spectroscopy. UV-Vis spectra of **FLDA** monomer shows absorption bands at λ_max_ = 445 nm and 373 nm (Figure 1A) corresponding to the transitions from the ground state (S_0_) to the S_1_ (λ_max_ ~ 442–450 nm) and S_2_ (λ_max_ ~ 360–375 nm) excited states (Heelis, 1982). These bands are red-shifted to ~ 456 nm for the S_0_ → S_1_ transition and ~ 376 nm for the S_0_ → S_2_ transition in **FLPDA** (Figure 1B), which can be explained by an increase in proton donation from PDA (Kotaki et al., 1970) and by electron-withdrawing inductive effects on the flavin moieties due to incorporation into the highly conjugated PDA system (Mataranga-Popa et al., 2015). The fluorescence emission spectra of **FLDA** and **FLPDA** are characterized by emission maxima at 527 nm (λ_ex_ = 450 nm), which correlates well to other known flavin compounds (Kotaki and Yagi, 1970), and confirms the presence of flavin moieties in **FLPDA** NPs (see Figures 1C,D). An earlier study in which flavin compounds were complexed with eumelanin showed that the fluorescent properties of flavins did not change upon binding and there is no significant fluorescence quenching by the polymer (Kozik et al., 1990). We have used this fact to approximate flavin concentration within **FLPDA** by means of a calibration curve comparison (see Figure S2 and Table S2). This gave an approximate concentration of flavin within **FLPDA** at 279.7 nmol/mg. It is worth noting that the amounts of flavin do not exactly match the monomer ratio used to synthesize the particles. The reason for this could be either the engulfing of the flavin moieties within particles or the base catalyzed cleavage/hydrolysis of flavin moieties during the polymerization, which results in the loss of characteristic flavin fluorescence (Smith and Bruice, 1975; Harayama et al., 1984).

**Figure 2:**
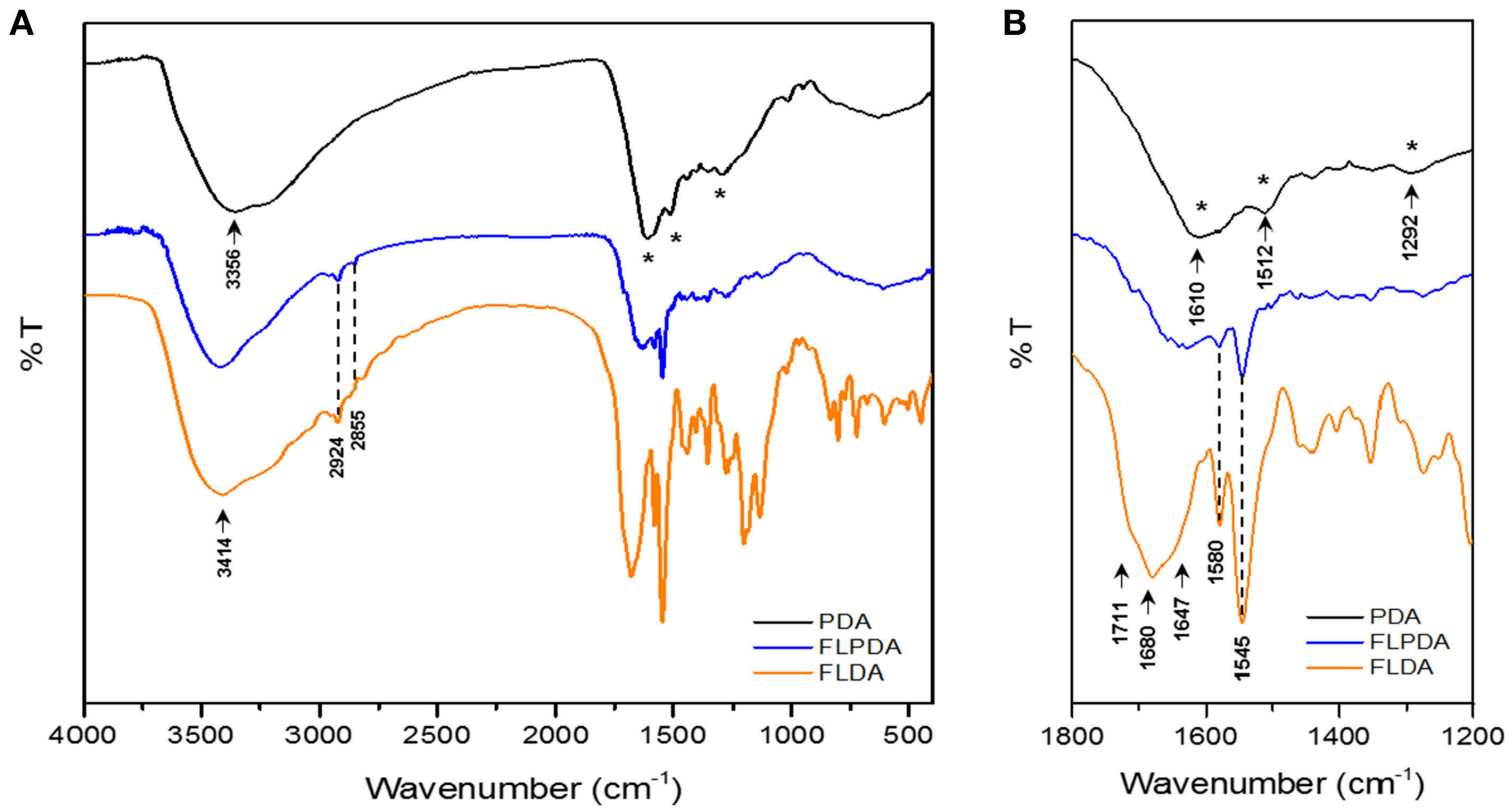
Full-scale FTIR **(A)** and zoomed **(B)** spectra of **PDA**, **FLPDA**, and **FLDA**.

FTIR spectroscopy was additionally employed to confirm **FLPDA** composition. The spectrum of PDA (Figure 2A) shows characteristic bands at 3,356 cm^−1^ relating to O-H and N-H stretching vibrations. In **FLDA** spectrum the bands corresponding to these vibrations broadens into one band at 3,414 cm^−1^, which is also the case for **FLPDA**. Unlike for PDA, in the spectra of **FLPDA** bands at 2,924 and 2,855 cm^−1^ corresponding to C-H stretching vibration can be clearly identified and correlated well to the spectrum of **FLDA**. As seen in the zoomed spectra in Figure 2B, bonds characteristic for flavins such as the sharp bands at 1,545 cm^−1^ and 1,580 cm^−1^ relating to ν(C = N) modes in the isoalloxazine ring (Rieff et al., 2011) can clearly be observed in FLPDA, This is also true for the contributions from flavin carbonyl ν(C = O) (1,711 and 1,680 cm^−1^) and ν(C = C). In addition, there are C = O and C-O vibrational modes of PDA, seen at 1,610 and 1,512 cm^−1^, respectively, which are not observed in FLDA. Further C = N and C = C combined contributions can also be seen at shifted wavenumbers in the spectrum of **FLPDA** (1,292 cm^−1^).

**Figure 3:**
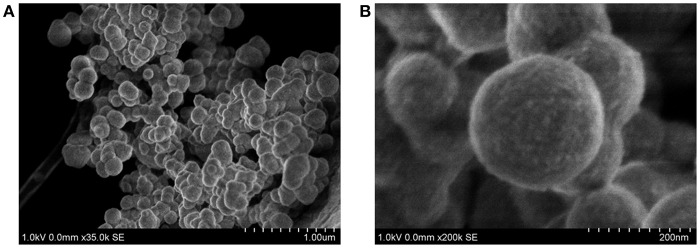
STEM images of **FLPDA** at different scales **(A)** scale bar = 1.00 μm and **(B)** scale bar = 200 nm.

Finally, the size and morphology of **FLPDA** were investigated using STEM. As shown in Figure 3 FLPDA solution is made up of spherical particles of similar size (~200 nm) with relatively large size distribution (± ~50 nm) (see Table S1). In contrast, PDA synthesized under the same conditions displays a narrower size distribution with an average size of 110 ± 18 nm (see Table S1), clearly indicating that the presence of flavin moieties affects the polymerization mechanism and oligomer aggregation to form particles with less defined shape and size. This is most likely due to H-bonding and electrostatic interactions between the flavin group and oligomeric units, and our ongoing work is focused on optimizing the polymerization procedure.

### Indole Photooxidation

**Figure 4:**
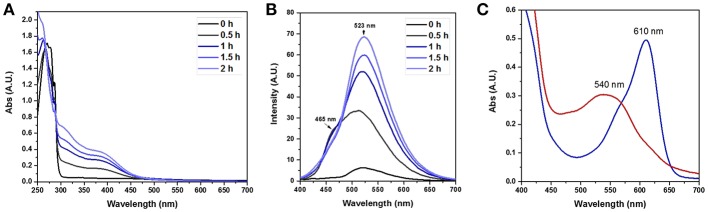
**(A)** UV-Vis absorption spectra of the photooxidation of indole (1.0 mM) in the presence of **FLPDA** (50 μg/mL) in O_2_ saturated ACN/H_2_O (1:1, v/v). **(B)** Fluorescence emission spectra (λ_ex_ = 380 nm) of indole (1.0 mM) photooxidation in the presence of **FLPDA** (50 μg/mL) in O_2_ saturated ACN/H_2_O (1:1, v/v). All spectra are 10× dilutions of reaction mixture. **(C)** UV-Vis absorption spectra of produced indirubin (λ_max_ = 540 nm) and indigo (λ_max_ = 610 nm) in DMF.

Having successfully prepared and characterized the **FLPDA** nanoparticles, we set out to investigate their catalytic activity in the presence of indole under blue light irradiation and explored their resemblance to the FMO enzyme activity. At first, small scale reactions were carried out using 1.0 mM indole with 50 μg/mL **FLPDA** and monitored by UV-Vis absorption spectroscopy. As shown in Figure 4A the absorption spectrum of indole (0 h) has a characteristic absorbance band at λ_max_ = 287 nm. After 0.5 h irradiation, this peak decreases in intensity and a new band appears at λ_max_ ~ 380 nm which is consistent with the consumption of indole and the production of 2 and 3-position hydroxylated indole species (Kumar and Kumar, 1998; Kuo and Mauk, 2012; Linhares et al., 2014). These bands increase in intensity with time and a shoulder replacing the characteristic band belonging to indole at 2 h indicates its consumption (Kuo and Mauk, 2012). We also monitored the fluorescence emission (λ_ex_ = 365 nm) of the reaction as shown in Figure 4B and observed the formation of fluorescent indoxyl species (λ_em_ = 465 nm) at 0.5 h and 1 h (Gehauf and Goldenson, 1957; Woo et al., 2000). After 1 h the signal disappears most likely due to indoxyl dimerization into the fluorescent, water-soluble leuco-indigo (λ_em_ = 523 nm) (Gehauf and Goldenson, 1957; Seixas De Melo et al., 2004). Control experiments were carried out in the dark showing no reaction after 18 h, and by irradiating indole alone, indole in the presence of pure PDA particles, and indole in the presence of **FLPDA** under inert Ar atmosphere; all of which showed negligible changes in indole's characteristic absorption band (see Figure S3).

**Figure 5:**
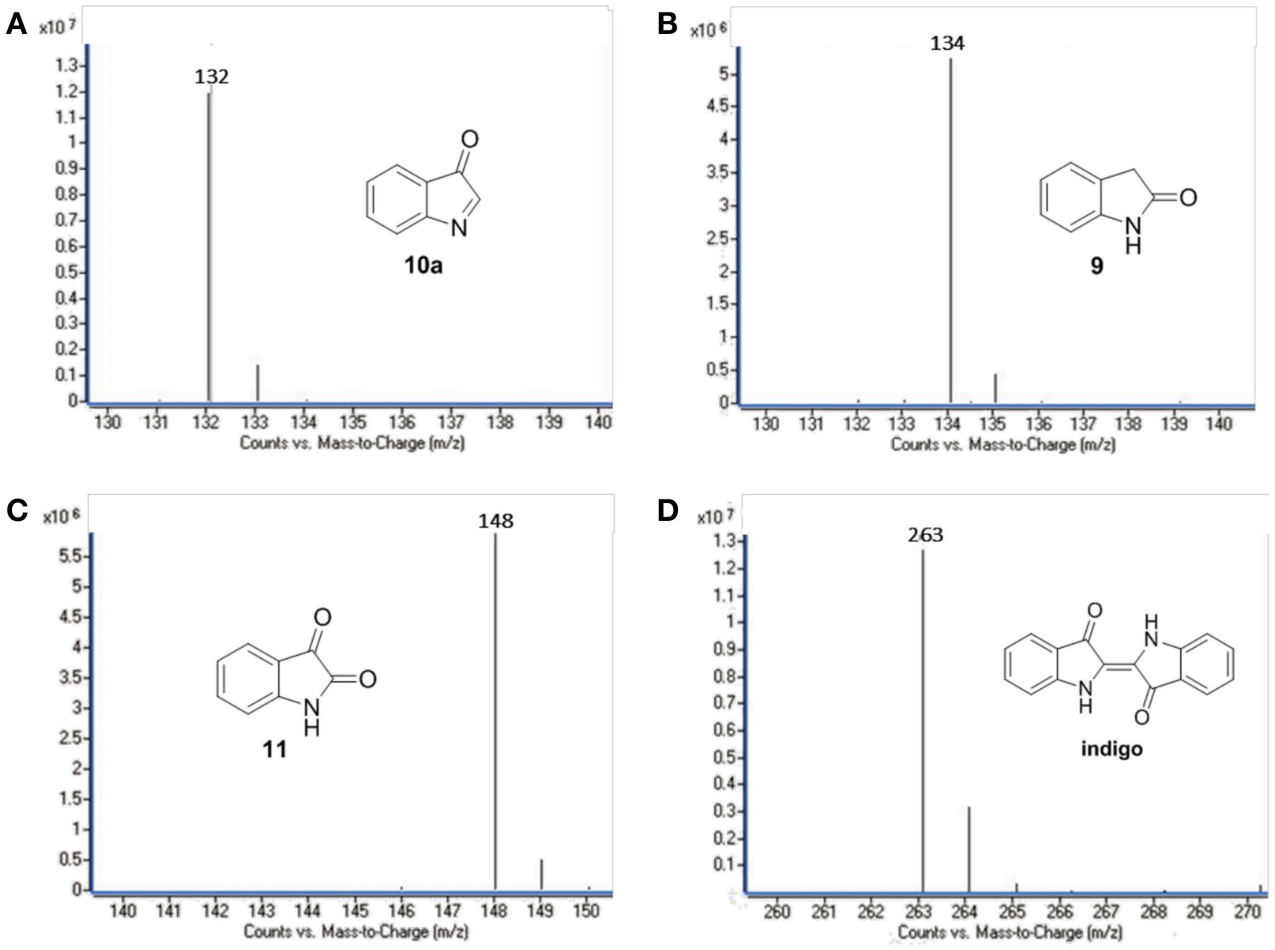
LC-MS chromatograms of the major oxidation products: **(A) 9**, **(B) 10a**, **(C) 11**, and **(D)** indigo.

**Scheme 4:**
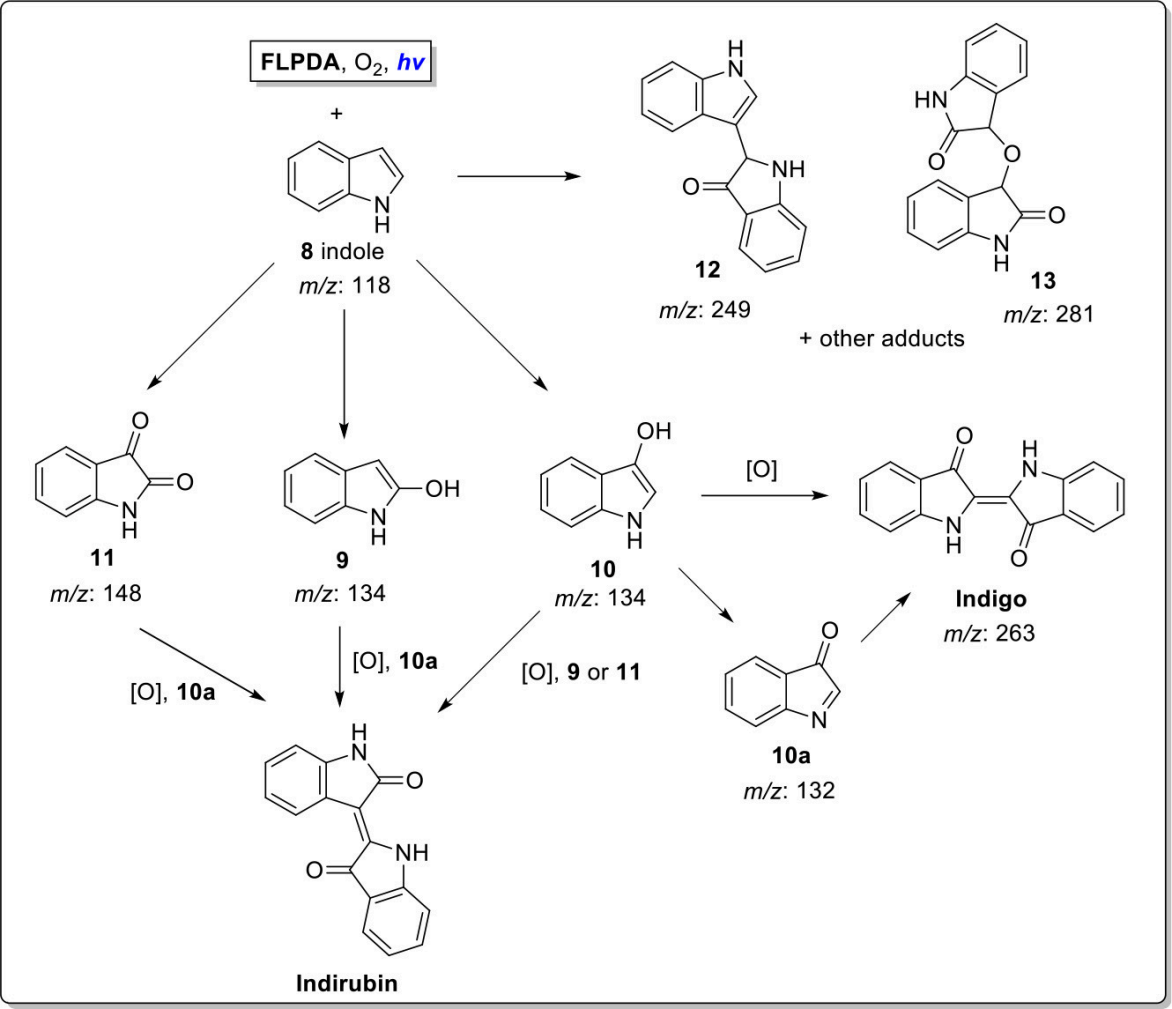
**Proposed reaction scheme for indole photooxidation in presence of enzyme mimicking **FLPDA****.

Several oxidation products were obtained from the **FLPDA** catalyzed photooxidation of indole and three major products were identified by LC-MS (Figure 5) and compared to the commercial reagents. These were isatin **11** (7.07 min, *m/z*: 148), 2-oxindole **9** (7.69 min, *m/z*: 134), and indoxyl **10** observed as its more stable keto-form **10a** (4.23 min, *m/z*: 132). Other products with higher *m/z* values were also identified *via* LC-MS of the reaction mixture, including indigo/indirubin (two signals with *m/z*: 263) and two others with m/z values of 249 and 281. Their structures are proposed in Scheme 4 and Figure S5 as products **12** and **13**, and their production is particularly interesting as they were observed in reactions catalyzed by enzymes such as laccases and P450s that contain inorganic cofactors (Gillam et al., 2000; Ganachaud et al., 2008; Linhares et al., 2014).

The formation of indigo and indirubin were initially observed by TLC analysis of the reaction mixture and were then isolated after reaction workup and characterized by UV-Vis (indirubin λ_max_ = 540 nm and indigo λ_max_ = 610 nm as seen in Figure 4C). It should be noted that theses dyes were not observed to form during the reaction itself, but only upon removal of the solvent to initiate precipitation or with the addition of weak acid to the reaction mixture or supernatant which catalyzed their formation (Zelentskii et al., 1970). Yields of the dyes were measured by UV-Vis spectroscopy using published extinction coefficients (Seixas De Melo et al., 2004) and were generally low (<4%) when precipitated out of solution after solvent removal however this is comparable to other biomimetic systems using Fe(II) and Mn(III) porphyrin complexes in the presence of H_2_O_2_ (Linhares et al., 2014; Rebelo et al., 2014) indicating that low yields are the consequence of the non-specific binding sites, as the active site of FMO alters the product selectivity (Han et al., 2012). We noticed that adding weak acid to the reaction mixture after irradiation provided better yields of dye, however, primarily favored the formation of indirubin (see Table 1).The light-driven oxidation of indole by **FLPDA** is clearly non-specific due to the range of identified oxidation products, however, these main products resemble those formed by FMOs and other xenobiotic degrading enzymes. To our knowledge, this is the only reported example of an organic nanoparticle-based photocatalyst that shows this activity. In addition, the only other example of photocatalytic oxidation of indole to form indigo and related compounds are CdS quantum dots, the use of which has serious implications in terms of toxicity (Tsoi et al., 2013; Yong et al., 2013).

**Table 1:** Amounts and conversions of major compounds identified by HPLC after acidification of reaction mixture using commercial standards as a calibration reference.

**Catalyst**	**Indole (μM)**	**Oxindole (9) (μM)**	**Isatin (11) (μM)**	**Indole conversion**	Indigoid yield^*^
FLPDA (36 ug/mL)	213	22	60	79%	5%
RF (20 μM)	24	16	80	97%	1%

* *([indigoid] × 2/[consumed indole]) (Xu et al., 2012) with [indigoid] being determined by UV-Vis spectroscopy*.

**Figure 6:**
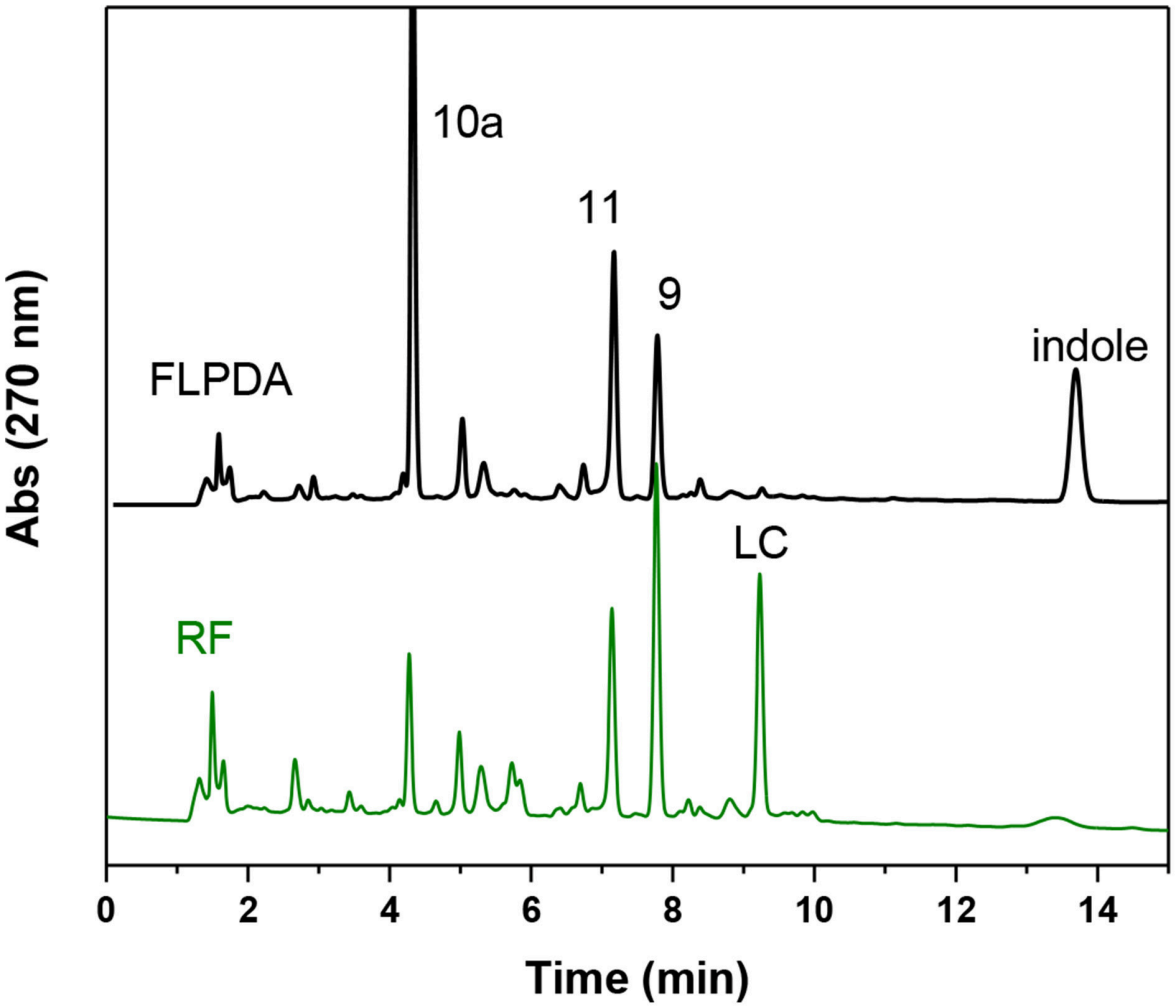
HPLC chromatograms of the reaction mixture after 2 h blue light irradiation of indole (1.0 mM) in the presence of **FLPDA** (36 μg/mL) in ACN/H_2_O (1:1, v/v) and riboflavin **RF** (20 μM).

**Figure 7:**
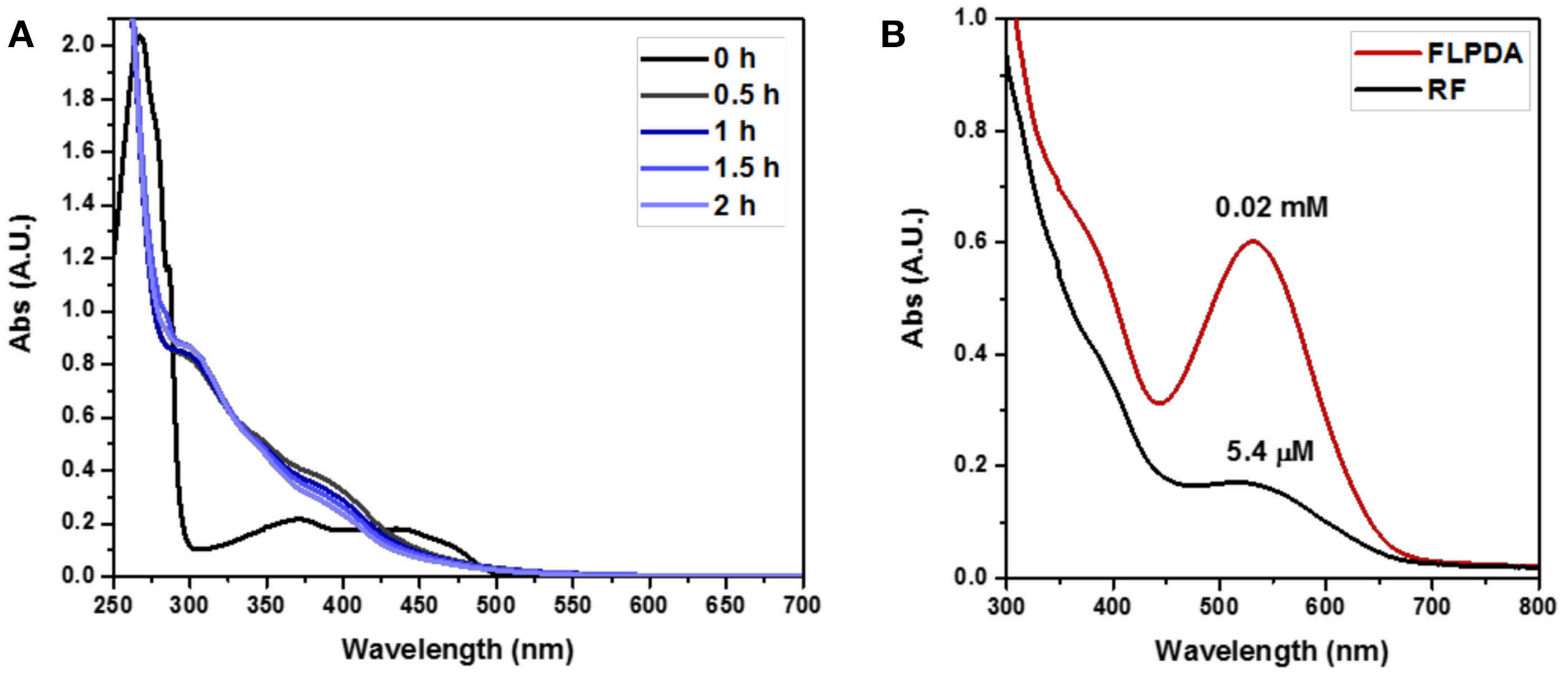
**(A)** UV-Vis absorption spectra of the photooxidation of indole (1.0 mM) in the presence of **RF** (20 μM) in O_2_ saturated ACN/H_2_O (1:1, v/v). **(B)** Plot comparing the reactions post acid workup (3× dilutions of reaction mixtures).

We also compared the activity of our hybrid catalyst to homogenous flavin, riboflavin (**RF**), under the same reaction conditions. To make an experimentally valid comparison, we used the same effective flavin concentration for both **FLPDA** and **RF** based on the fluorescence calibration curve utilized to characterize **FLPDA**, namely 20 μM for **RF** (2 mol%) and 36 μg/mL **FLPDA**. UV-Vis analysis of the reaction mixture containing **RF** showed an increased rate of indole consumption (shoulder formation at ~280 nm as shown in **Figure 7A**) after 0.5 h. HPLC analysis indicated that there is a greater conversion of indole in presence of RF compared to **FLPDA**, and similar products although in different yields, were observed as shown in Figure 6 and Table 1. We next compared the yield of dyes after the reactions through the addition of 2 drops of 1M HCl to each reaction mixture to initiate dimerization of hydroxylated indole species to form the indigoid dyes. As shown in Figure 7B, there was more indirubin obtained from the reaction conducted in the presence of **FLPDA** than **RF**, which was expected as 2.5 times more precursor **10a** can be detected in HPLC profile shown in Figure 6. Clearly, despite the decreased indole conversion, **FLPDA** appears to be more selective than its homogeneous counterpart toward dye conversion.

It is also worth noting that after the irradiation, **RF** could no longer be identified by TLC analysis within the reaction mixture and had degraded to lumichrome (LC), which was confirmed both by HPLC and LC-MS (9.22 min, *m/z*: 243) (Figure 6). Although there was some photodecomposition observed in **FLPDA**, the concentration of lumichrome was five times lower compared to **RF** (based on HPLC integration), indicating that the hybrid catalyst offers more protection toward photodecomposition of active flavin centers, and mitigates the loss of catalysis observed in homogenous systems.

### Further Control Experiments and Possible Mechanism of Action

**Figure 8:**
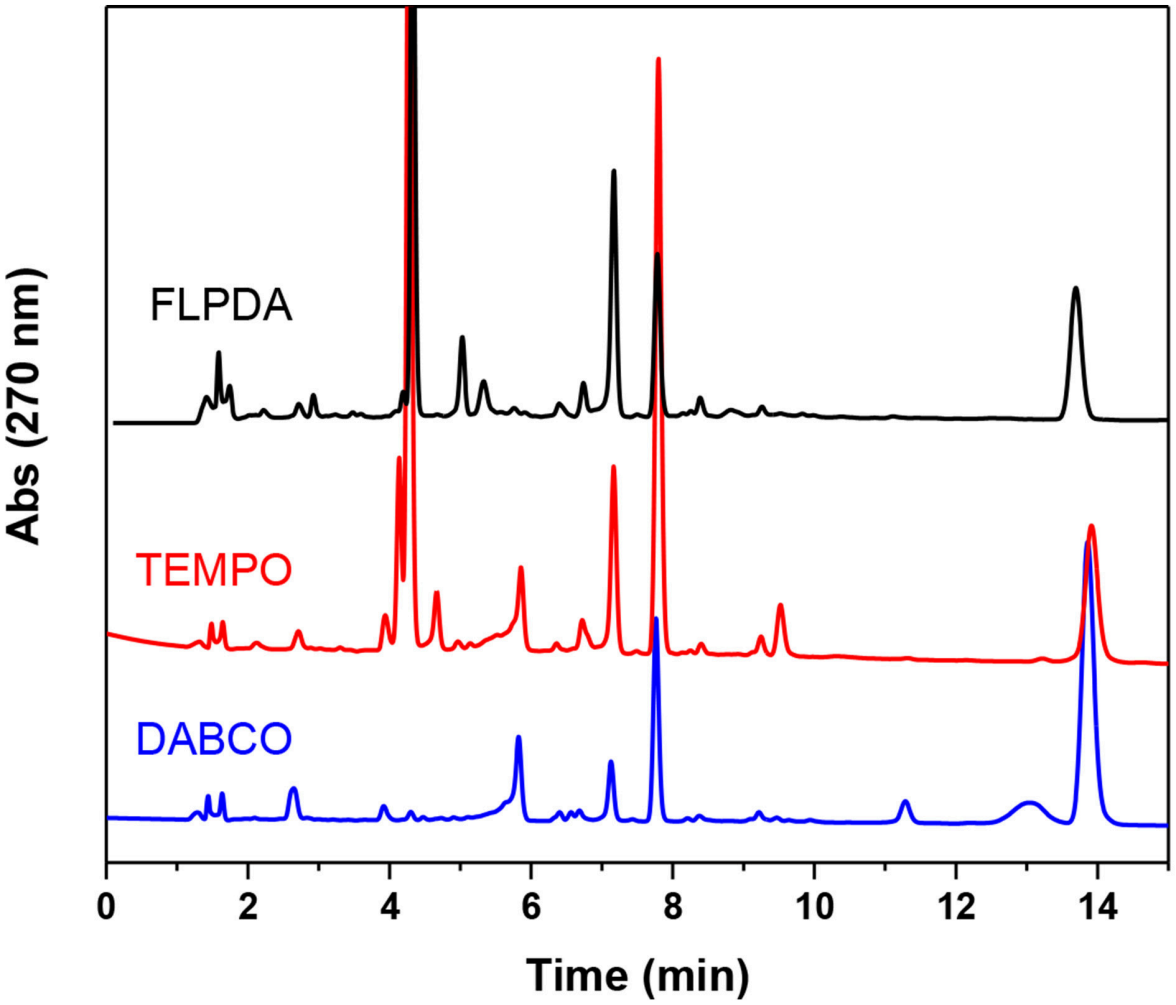
HPLC chromatograms of control experiments in which **FLPDA** (50 μg/mL) and indole (1.0 mM) were irradiated in presence of radical scavengers DABCO (1.0 mM) and TEMPO (1.0 mM).

As this is a novel hybrid system, we were also interested in unveiling the photocatalytic mechanism of **FLPDA**. To achieve this we employed a superoxide radical scavenger TEMPO and the singlet oxygen scavenger DABCO to quench these prominent reactive oxygen species (ROS) that may be liberated from reduced and/or photoexcited flavin species (Massey et al., 1969; Müller and Ahmad, 2011). As can be seen from the HPLC chromatograms of the reactions shown in Figure 8, the addition of these quenchers did not appear to inhibit the reaction completely. In fact, we observed that in the case of DABCO, the basic character of the species may have played a role in lowering the activity of **FLPDA** as flavins and PDA are less stable within a basic environment (Song et al., 1965; Yang et al., 2018). In terms of product selectivity, **10a** was not detected most likely due to direct oxidization to isatin **11** in basic condition (Table 2). Based on these observations it could be assumed that singlet oxygen plays a minimal role in **FLPDA** activity as both compounds **9** and **11** are obtained in the presence of singlet oxygen quencher.

**Table 2:** Amounts and conversions of major compounds identified by HPLC after acidification of reaction mixture using commercial standards as a calibration reference.

**Quencher**	**Indole (μM)**	**Oxindole (9) (μM)**	**Isatin (11) (μM)**	**Indole conversion**
TEMPO	177	278	121	82%
DABCO	326	143	252	67%

The addition to the superoxide scavenger, TEMPO had no deleterious effect on **FLPDA** activity either and in fact, it enhanced the production of **9, 10a**, and **11**. In fact, TEMPO most probably acts as a redox mediator and co-catalyst in the reaction, as previously shown for the synthesis of isatin derivatives in the presence of hypervalent iodine (Sai Prathima et al., 2015). TEMPO's participation in the reactions was additionally proved by appearance of the by-product 2,2,6,6-tetramethylpiperidine (TMP) at a retention time of 2.49 min, *m/z*: 142 (LC-MS analysis). It should be noted that pigment melanin, composed of PDA units, is capable of superoxide quenching (Tada et al., 2010), and it is safe to assume that any superoxide radical generated by flavin would be quickly quenched by PDA in its immediate proximity.

**Scheme 5:**
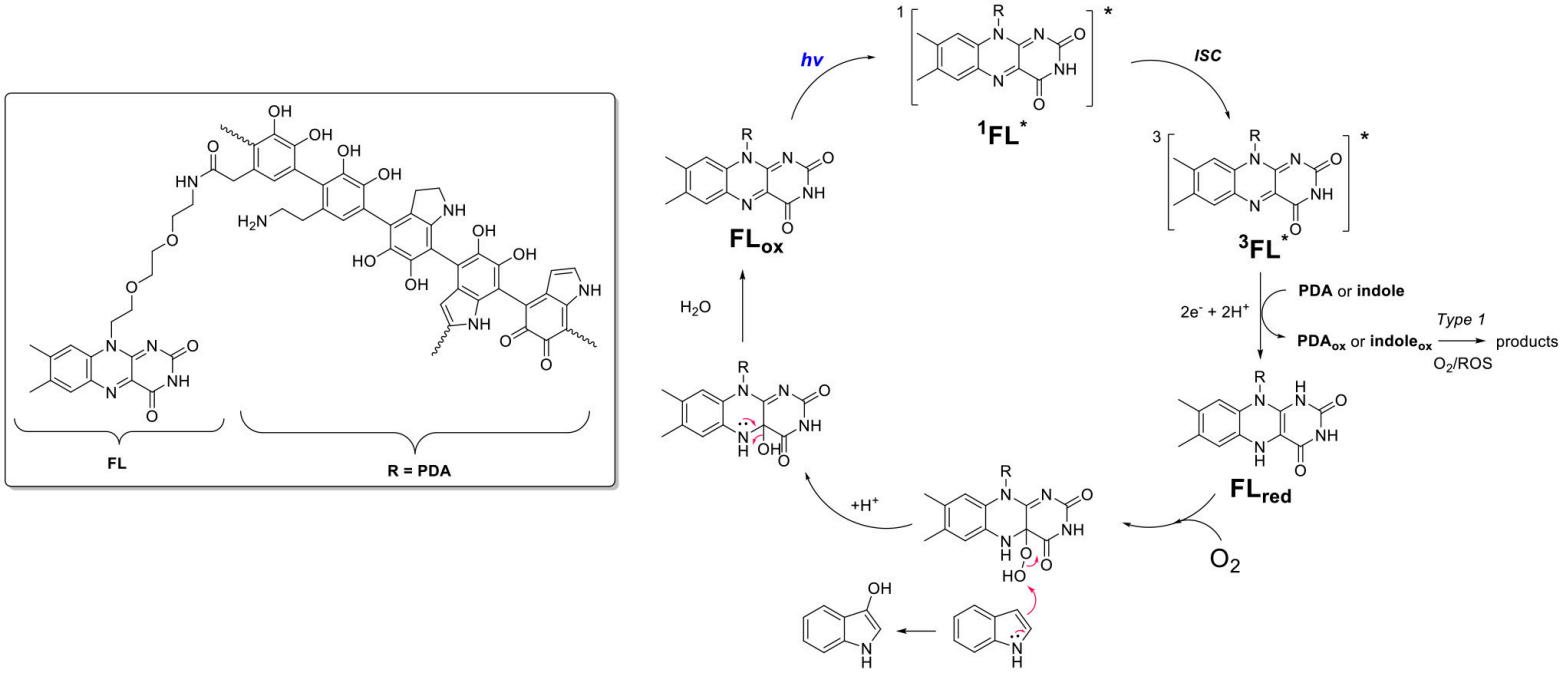
**Proposed mechanism of action involving electron transfer process between flavin (FL) and PDA moieties within **FLPDA** hybrid catalyst**.

We further investigated the mechanism of **FLPDA**'s photocatalytic activity by using a fluorescence-based assay to monitor the release of H_2_O_2_ liberated from the unstable 4a-hydroperoxy-flavin species formed when reduced flavin moieties interact with oxygen (Usselman et al., 2014). We reasoned that the lower amount of H_2_O_2_ obtained in photo-reaction in presence of **FLPDA** compared to free flavin (riboflavin **RF)**, would indicate potential stabilization of 4a-hydroperoxy-flavin species, and its interaction with indole (Scheme 5) instead of direct transformation into reactive oxygen species. To minimize the loss of photons due to the scattering effect of a colloidal heterogeneous catalyst, low concentration of **FLPDA** (10 μg/mL) was used, and the concentration of indole and effective concentration of free flavin were adjusted accordingly. The amount of H_2_O_2_ released after 1 h of irradiation was 3.62 ± 0.18 nmol for **FLPDA** and 5.13 ± 0.04 nmol for **RF** (see Figure S4). The lower value obtained for **FLPDA** suggest that the stabilization of 4a-hydroperoxy flavin species in **FLPDA** may occur, although this hypothesis can only be fully confirmed after the completion of already initiated EPR study.

Based on current data, we propose the mechanism shown in Scheme 5, which includes the formation of the 4a-hydroperoxy species as a result of indole and/or PDA photoreduction, and subsequent interaction with oxygen. This species could be stabilized by H-bonding with neighboring PDA moieties and be stable long enough to interact with the indole substrate. In addition, the appearance of compounds **12** and **13** indicates Type 1 photosensitization of indole through electron transfer to excited flavin moieties similar to that previously observed for riboflavin and tryptophan (Silva et al., 2019). However, as stated above further studies using EPR spectroscopy are being undertaken to confirm our theory.

Although some “dark” reactions in the presence of flavin species have resulted in excellent yields of desired products (Arakawa et al., 2017; Chevalier et al., 2018), use of light to trigger the reaction cascade that leads to complex dye formation would have multiple advantages in an industrial setting, including temporal and spatial control, ultimately leading to the design of more efficient reactors.

### Recyclability

**Figure 9:**
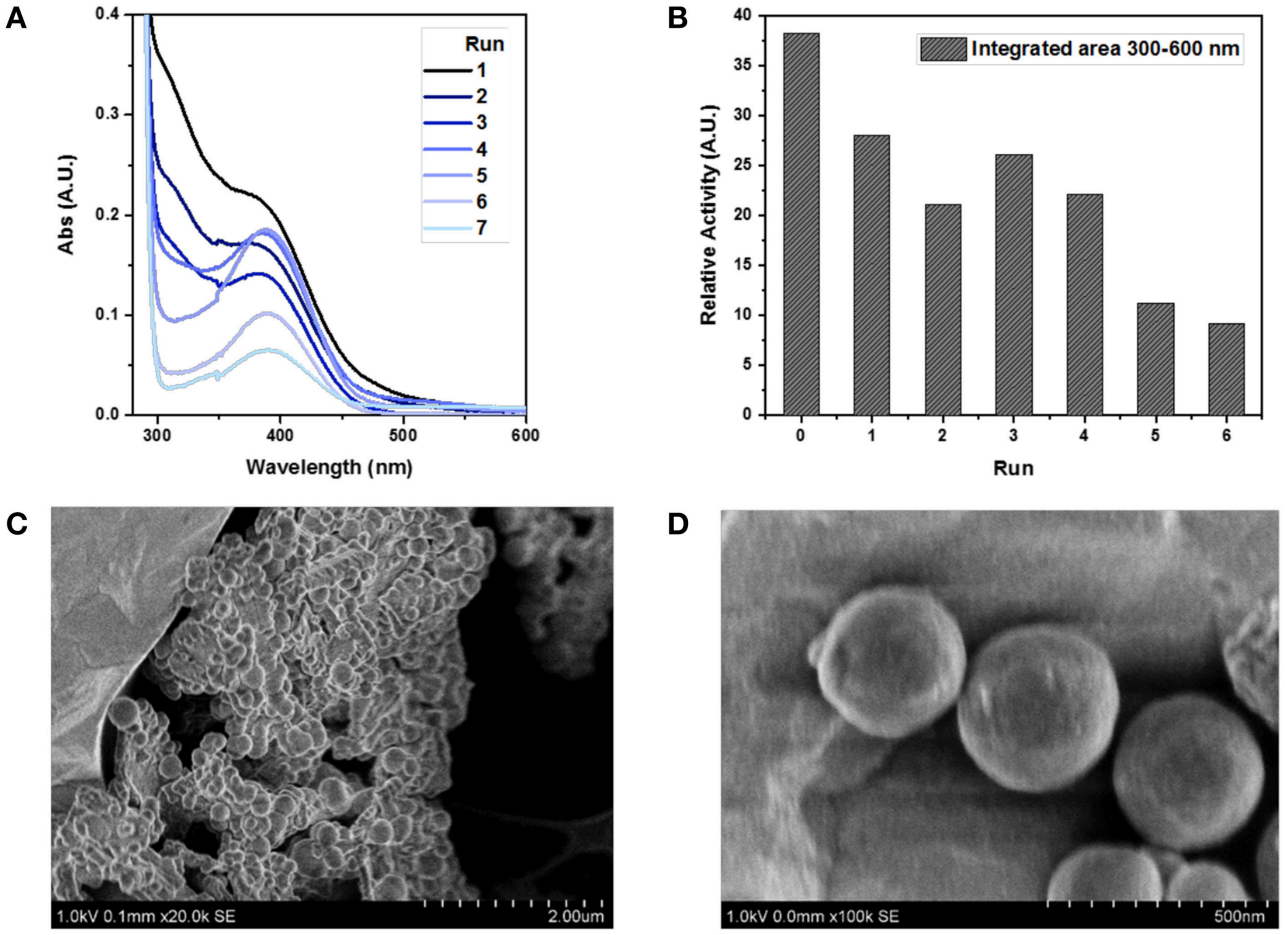
Study of FLPDA catalyst recyclability. UV-Vis absorption spectra of different runs using a recycled catalyst **(A)** and the relative activity plot using the integrated area between 300 and 600 nm of each run **(B)**. STEM image of FLPDA particles after 2 h irradiation shown at different scales **(C)** scale bar = 2.00 μm, and **(D)** scale bar = 500 nm. Reaction conditions: indole (1.0 mM), **FLPDA** (50 μg/mL) in O_2_ saturated ACN/H_2_O (1:1, v/v) and irradiation for 1.5 h.

Finally, we were interested to estimate the reusability of **FLPDA** in this light-driven reaction as this is a desirable property for scaling up and industrial applications enhancing the green potential of the system. Previous work on a heterogeneous flavin-based photocatalytic system using mesoporous silica to immobilize flavin moieties suffered from a severe loss of activity after the first reuse, and a complete inactivation upon second and third attempt (Špačková et al., 2017). For our nanoparticle-based system, the catalyst was easily removed from the reaction mixture *via* centrifugation and maintained activity for 4 cycles. As a proof of concept study, activity was monitored by irradiation of **FLPDA** particles (50 μg/ml) with indole (1.0 mM) for 1.5 h and measuring the changes in absorption of the reaction mixture between 300 and 600 nm as shown in Figure 9A. The area within this region was integrated and used as a measure for oxidized indole species production. The first run showed a decrease in relative activity compared to the initial run only by a factor of 1.36 (Figure 9B) and the subsequent three runs stayed within a similar range before minimum activity, although not a complete loss, was observed by run 6.

The enhanced stability of our system could be explained by the electron transfer from PDA to flavin moieties upon irradiation as proposed in our mechanism (Scheme 5). It has already been shown that an addition of an electron donor, such as 2-(*N*-morpholino)ethanesulfonic acid (MES) buffer, limits photo-induced decomposition reactions that curtail flavin activity upon irradiation (Alonso-De Castro et al., 2017). However, during the recycling steps, the number of retrieved particles decreased with each run. Loss of particles and the catalytic activity could point toward the photo-degradation of PDA moieties similar to what was previously observed in melanin samples (Ito et al., 2018). However, we did not notice any difference in the particle size and morphology after 2 h of irradiation (Figures 9C,D), which disputes this argument, and indicates a mere physical loss of particles during recovery procedures.

Our ongoing work is focused on exploring the long-term stability and photo-degradation of **FLPDA**, as well as establishing a detailed mechanism using EPR studies. We believe this information will allow us to rationally design better catalytic systems with enantio-selective potential. We are also currently working on improving the reusability of **FLPDA** by addition of magnetic Fe_3_O_4_ nanoparticles during co-polymerization to enable magnetically aided retrieval, which has already proven effective in various application using PDA (Liu et al., 2013, 2017; Xie et al., 2014; Yu et al., 2016).

## Conclusion

We have developed a novel photocatalytic organic nanoparticle system made from flavin conjugated polydopamine. This was achieved through a convenient co-polymerization method involving dopamine and a flavin-dopamine analog, **FLDA**, to form **FLPDA**. The system effectively displays activity analogous to xenobiotic-degrading enzymes such as FMO, shown, using the oxidation reaction of indole, to form the indigo dye. In addition, it employs irradiation with visible light rather than an external cofactor for catalyst activation. The products observed in the reaction; indigo, indirubin, and related compounds, clearly indicated FMO-like activity of **FLPDA**. Additional ROS scavenging studies as well as the nature of some products, further confirmed that **FLPDA** displays mechanistic similarities to natural FMO, which is characterized by the formation of 4a-hydroperoxy flavin as a reactive intermediate. In contrast to our heterogeneous hybrid flavin, free flavin in a solution was characterized with higher photo-degradation and lower dye yields. In addition, **FLPDA** can easily be recovered from the reaction mixture and displays recyclable activity up to four cycles. We hope that this initial work sets the foundation to enable the design of more elaborate photocatalytic biopolymer hybrids comprising of flavin, PDA and additional functional moieties, to be applied in heterogeneous catalysis and artificial photosynthesis.

## Author Contributions

LC carried out all the synthesis, characterization and analyses, and wrote the draft manuscript. LF was involved in the experimental planning and manuscript proofreading and corrections. LF was the academic lead of the project in charge of experimental planning and funding.

### Conflict of Interest Statement

The authors declare that the research was conducted in the absence of any commercial or financial relationships that could be construed as a potential conflict of interest.
