# Supplementary material for: Flavin Conjugated Polydopamine Nanoparticles Displaying Light-Driven Monooxygenase Activity
Source: Front Chem. 2019 Apr 26;7:278. doi: 10.3389/fchem.2019.00278 (PMC6497766; doi:10.3389/fchem.2019.00278)
Supplement: Supplementary file 1 [file Table_1.pdf]

Supplementary Material

1 Synthesis of Flavin-Dopamine Monomer

N^1-(2-(2-(2-azidoethoxy)ethoxy)ethyl)-4,5-dimethylbenzene-1,2-diamine (2):

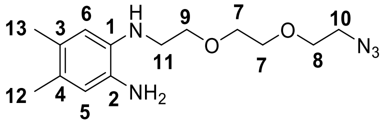

1-Azido-2-(2-(2-iodoethoxy)ethoxy)ethane (**1**) was synthesised according to the literature procedure by Deng *et al.* [1]

4,5-Dimethylbenzene-1,2-diamine (1.00 g, 7.34 mmol) and K_2CO_3 (2.03 g, 14.68 mmol) were dissolved in anhydrous DMF (30 mL) and heated to 50 ^oC under Ar atmosphere. 1-Azido-2-(2-(2-iodoethoxy)ethoxy)ethane (2.09 g, 7.34 mmol) dissolved in anhydrous DMF (10 mL) was then added dropwise and the resulting mixture was stirred at 50 ^oC overnight. DMF was removed under reduced pressure and the resulting residue was re-dissolved in DCM (50 mL) and washed with water (3 x 50 mL) and brine (2 x 50 mL). The organic layer was then dried over anhydrous Na_2SO_4 and evaporated. The crude product was then purified by column chromatography using the solvent system DCM/MeOH (99.5:0.5) to give the title compound as a red oil (1.50 g, 5.11 mmol, 70%).

^1H NMR (500 MHz, $CDCl_3$): δ = 6.52 (s, 1H, **H-5**), 6.48 (s, 1H, **H-6**), 3.74 (t, 2H, ^3J = 5.2 Hz, **H-9**), 3.70-3.67 (m, 6H, **H-7**, **H-9**), 3.39 (t, 2H, ^3J = 5.1 Hz, **H-10**), 3.27 (br. s, 2H, **H-11**), 2.15 (2 x br. s, 6H, **H-12**, **H-13**) ppm

^{13}C NMR (500 MHz, $CDCl_3$): δ = 134.91 (**C-1**), 132.93 (**C-2**), 127.72 (**C-3**), 126.90 (**C-4**), 118.24 (**C-5**), 115.11 (**C-6**), 70.68 (**C-7**), 70.35 (**C-8**), 70.11 (**C-9**), 50.71 (**C-10**), 44.45 (**C-11**), 19.24 (**C-12**), 18.84 (**C-13**) ppm

HRMS (ESI) m/z: $[M + H]^+$ Calcd for $C_{14}H_{24}O_2N_5$ 294.1925; Found 294.1914.

10-(2-(2-(2-Azidoethoxy)ethoxy)ethyl)-7,8-dimethylbenzo[g]pteridine-2,4(3H,10H)-dione (3):

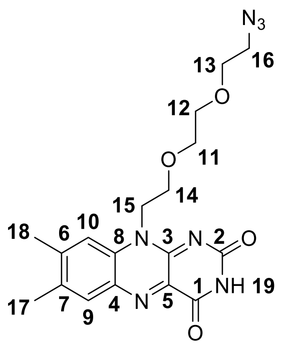

N[1]-(2-(2-(2-azidoethoxy)ethoxy)ethyl)-4,5-dimethylbenzene-1,2-diamine (**2**) (2.50 g, 8.52 mmol), B_2O_3 (1.19 g, 17.04 mmol) and alloxan monohydrate (1.21 g, 8.52 mmol) were dissolved in glacial acetic acid (25 mL) and left to stir in the dark at room temperature under Ar atmosphere for 2 days. Water was added (25 mL) and extracted with DCM (3 x 50 mL). The organic layer was evaporated and then co-evaporated with toluene (3 x 50 mL) to remove any traces of water and acetic acid. The crude product was purified by column chromatography using a gradient solvent system of DCM/acetone (4:1 - 1:1) to give the title compound as an orange solid (2.35 g, 5.89 mmol, 69%).

^{1}H NMR (500 MHz, DMSO-d$_6$): δ =11.30 (s, 1H, **H-19**), 7.82 (s, 1H, **H-9**), 7.81 (s, 1H, **H-10**), 4.76 (t, 2H, ^{3}J = 5.8 Hz, **H-15**), 3.81 (t, 2H, ^{3}J = 5.8 Hz, **H-14**), 3.54 (m, 2H, **H-13**), 3.45 (m, 4H, **H-11**, **H-12**), 3.25 (t, 2H, ^{3}J = 5.8 Hz, **H-16**), 2.47 (s, 1H, **H-17**), 2.36 (s 1H, **H-18**) ppm

^{13}C NMR (500 MHz, DMSO-d$_6$): δ = 160.33 (**C-1**), 155.98 (**C-2**), 150.66 (**C-3**), 146.65 (**C-4**), 137.48 (**C-5**), 136.20 (**C-6**), 134.10 (**C-7**), 131.83 (**C-8**), 131.18 (**C-9**), 117.30 (**C-10**), 70.67 (**C-11**), 70.06 (**C-12**), 69.73 (**C-13**), 67.24 (**C-14**), 50.39 (**C-15**), 44.56 (**C-16**), 21.06 (**C-17**), 19.22 (**C-18**) ppm

HRMS (ESI) m/z: $[M + Na]^+$ Calcd for $C_{18}H_{21}O_4N_7Na$ 422.1547; Found 422.1528.

10-(2-(2-(2-aminoethoxy)ethoxy)ethyl)-7,8-dimethylbenzo[g]pteridine-2,4(3H,10H)-dione (4):

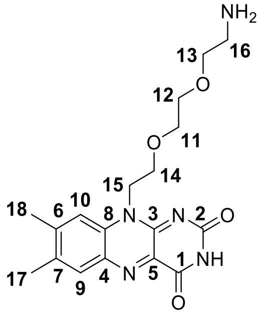

10-(2-(2-(2-Azidoethoxy)ethoxy)ethyl)-7,8-dimethylbenzo[g]pteridine-2,4(3H,10H)-dione (**3**) (0.50 g, 1.25 mmol) was added to a dried 100 mL 3 neck round bottom flask and dissolved in degassed glacial acetic acid (50 mL). The vessel was purged with Ar by water aspiration before adding Pd/C (10 mg), rinsing any residual powder off of the flask walls with degassed acetic acid. The vessel was purged with Ar again before replacing the atmosphere with H_2 from a balloon. The reaction was then left to stir for 18 h at room temperature. The atmosphere was then replaced by Ar and the reaction mixture filtered through celite, washed with methanol and evaporated. The crude residue was subjected to flash column chromatography using the solvent system DCM/MeOH/AcOH (70:20:10 - 20:70:10) to give the title compound as a red residue (0.43 g, 1.05 mmol, 84%).

^{1}H NMR (500 MHz, CD_3OD): δ = 8.20 (s, 1H, **H-9**), 8.14 (s, 1H, **H-10**), 5.17 (t, 2H, ^{3}J = 5.0 Hz, **H-15**), 4.13 (t, 2H, ^{3}J = 5.0 Hz, **H-14**), 3.68 (m, 2H, **H-13**), 3.64 – 3.59 (m, 4H, **H-11**, **H-12**), 3.08 (t, 2H, ^{3}J = 5.0 Hz, **H-16**), 2.68 (s, 3H, **H-17**), 2.55 (s, 3H, **H-18**) ppm

^{13}C NMR (500 MHz, CD_3OD): δ = 158.9 (**C-1**), 151.6 (**C-2**), 150.8 (**C-3**), 145.9 (**C-4**), 140.6 (**C-5**), 137.1 (**C-6**), 134.4 (**C-7**), 131.3 (**C-8**), 130.5 (**C-9**), 117.3 (**C-10**), 70.4 (**C-11**), 69.8 (**C-12**), 67.7 (**C-13**), 66.4 (**C-14**), 42.8 (**C-15**), 39.2 (**C-16**), 20.3 (**C-17**), 18.2 (**C-18**) ppm

HRMS (ESI) m/z: $[M + H]^+$ Calcd for $C_{18}H_{24}O_4N_5$ 374.1823; Found 374.1828.

2-(2,2-Dimethylbenzo[d][1,3]dioxol-5-yl)acetyl chloride (5):

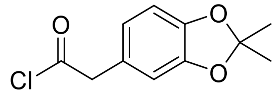

2-(2,2-Dimethylbenzo[d][1,3]dioxol-5-yl)acetic acid was synthesised according to the literature procedure by Geiseler *et al.*[2]

2-(2,2-Dimethylbenzo[d][1,3]dioxol-5-yl)acetic acid (0.42 g, 2.02 mmol) was dissolved in anhydrous DCM (40 mL) before adding $SOCl_2$ (1.46 mL, 20.17 mmol) and refluxing for 2 h under Ar atmosphere. The reaction mixture was evaporated to remove the solvent and excess $SOCl_2$ was removed by toluene co-evaporation (3 x 20 mL). The resulting dark brown oil was immediately used in the following step without further purification (0.46 g, 2.02 mmol, 100%).

N-(2-(2-(2-(7,8-Dimethyl-2,4-dioxo-3,4-dihydrobenzo[g]pteridin-10(2H)-yl)ethoxy)ethoxy)ethyl)-2-(2,2-dimethylbenzo[d][1,3]dioxol-5-yl)acetamide (6):

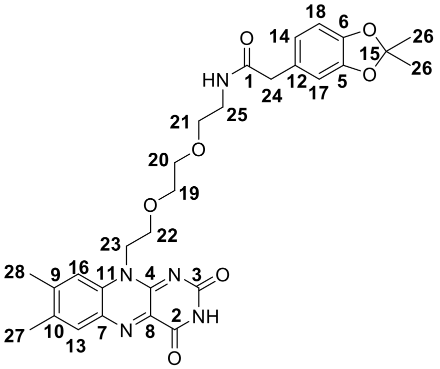

10-(2-(2-(2-aminoethoxy)ethoxy)ethyl)-7,8-dimethylbenzo[g]pteridine-2,4(3H,10H)-dione (**4**) (0.40 g, 0.98 mmol) was dissolved in anhydrous DMF (40 mL) under Ar atmosphere and protected from light. Et_3N (0.27 mL, 1.95 mmol) was added and the solution turned form dark red to dark green. The mixture was then cooled to 0 ^oC before adding 2-(2,2-dimethylbenzo[d][1,3]dioxol-5-yl)acetyl chloride (0.44 g, 1.95 mmol) dissolved in anhydrous DMF (10 mL) dropwise over 20 minutes. The resulting dark red reaction mixture was left to stir and warm to room temperature over 18 h. The solvent was then removed and the crude residue purified by column chromatography using the solvent system DCM/MeOH/AcOH (98.5:1:0.5 – 90:6:4) to give the title compound as an orange solid (0.355 g, 0.63 mmol, 64%).

^1H NMR (500 MHz, CD_3OD): δ = 7.94 (s, 1H, **H-13**), 7.91 (s, 1H, **H-16**), 6.64 (m, 2H, **H-17**, **H-18**), 6.59 (d, 1H, ^3J = 8.1 Hz, **H-14**), 4.97 (t, 2H, ^3J = 5.4 Hz, **H-23**), 3.98 (t, 2H, ^3J = 5.4 Hz, **H-22**), 3.58 (m, 2H, **H-19**), 3.46 (m, 2H, **H-20**), 3.36 (t, 2H, ^3J = 5.6 Hz, **H-21**), 3.34 (s, 2H, **H-24**) 3.21 (t, 2H, ^3J = 5.6 Hz, **H-25**), 2.56 (s, 3H, **H-27**), 2.46 (s, 3H, **H-28**), 1.58 (s, 6H, **H-26**) ppm

^{13}C NMR (500 MHz, CD_3OD): δ = 173.0 (**C-1**), 157.8 (**C-2**), 150.6 (**C-3**), 147.8 (**C-4**), 147.5 (**C-5**), 146.4 (**C-6**), 137.5 (**C-7**), 137.2 (**C-8**), 136.2 (**C-9**), 135.7 (**C-10**), 134.8 (**C-11**), 132.2 (**C-12**), 130.9 (**C-13**), 121.3 (**C-14**), 117.6 (**C-15**), 117.1 (**C-16**), 108.7 (**C-17**), 107.5 (**C-18**), 70.5 (**C-19**), 69.9 (**C-**

20), 69.0 (**C-21**), 67.5 (**C-22**), 45.0 (**C-23**), 42.1 (**C-24**), 39.0 (**C-25**), 24.5 (**C-26**), 20.0 (**C-27**), 18.0 (**C-28**) ppm

HRMS (ESI) m/z: [M + H]+ Calcd for $C_{29}H_{34}O_7N_5$ 564.2453; Found 564.2448.

2-(3,4-Dihydroxyphenyl)-N-(2-(2-(2-(7,8-dimethyl-2,4-dioxo-3,4-dihydrobenzo[g]pteridin-10(2H)-yl)ethoxy)ethoxy)ethyl)acetamide (FLDA):

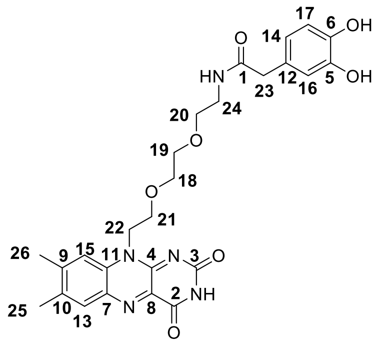

N-(2-(2-(2-(7,8-Dimethyl-2,4-dioxo-3,4-dihydrobenzo[g]pteridin-10(2H)-yl)ethoxy)ethoxy)ethyl)-2-(2,2-dimethylbenzo[d][1,3]dioxol-5-yl)acetamide (**5**) (0.20 g, 0.35 mmol) was dissolved in DCM (20 mL) and TFA (4 mL) was added dropwise at 0 ^{o}C. The reaction mixture was then left to stir at room temperature until TLC analysis showed completion (2 h). The solvent and excess TFA were removed under reduced pressure and co-evaporated with toluene (3 x 20 mL) to give the title compound as a red solid (0.184 g, 0.35 mmol, 99%).

^{1}H NMR (500 MHz, CD_3OD): δ = 7.84 (s, 1H, **H-13**), 7.82 (s, 1H, **H-15**), 6.67 (d, 1H, ^{4}J = 2.1 Hz, **H-16**), 6.64 (d, 1H, ^{3}J = 8.0 Hz, **H-17**), 6.54 (dd, 1H, ^{3}J = 8.0 Hz, ^{4}J = 2.1 Hz, **H-14**), 4.90 (t, 2H, ^{3}J = 5.5 Hz, **H-22**), 3.94 (t, 2H, ^{3}J = 5.5 Hz, **H-21**), 3.55 (m, 2H, **H-18**), 3.44 (m, 2H, **H-19**), 3.36 (t, 2H, ^{3}J = 5.5 Hz, **H-20**), 3.29 (s, 2H, **H-23**) 3.21 (t, 2H, ^{3}J = 5.5 Hz, **H-24**), 2.52 (s, 3H, **H-25**), 2.42 (s, 3H, **H-26**) ppm

^{13}C NMR (500 MHz, CD_3OD): δ = 174.8 (**C-1**), 162.3 (**C-2**), 151.7 (**C-3**), 149.4 (**C-4**), 146.4 (**C-5**), 145.3 (**C-6**), 138.9 (**C-7**), 138.7 (**C-8**), 137.3 (**C-9**), 136.1 (**C-10**), 133.5 (**C-11**), 132.2 (**C-12**), 128.2 (**C-13**), 121.5 (**C-14**), 118.5 (**C-15**), 117.2 (**C-16**), 116.4 (**C-17**), 71.9 (**C-18**), 71.3 (**C-19**), 70.5 (**C-20**), 68.8 (**C-21**), 46.4 (**C-22**), 43.3 (**C-23**), 40.4 (**C-24**), 21.4 (**C-25**), 19.4 (**C-26**) ppm

HRMS (ESI) m/z: [M + H]+ Calcd for $C_{26}H_{29}O_7N_5Na$ 546.1959; Found 546.1948.

2 LED strips reactor setup

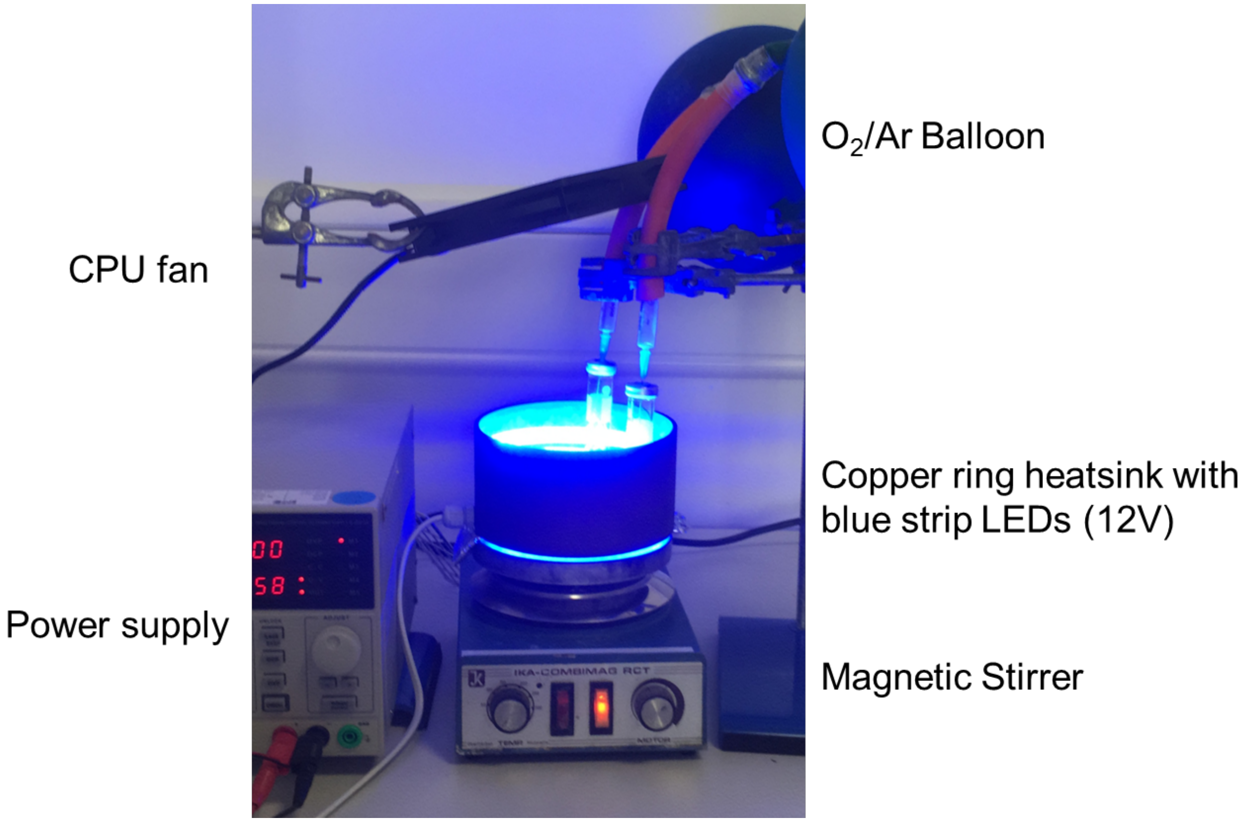

Figure S1: The setup was built drawing influence from the LED strips setup described by the MacMillan group.[3] Blue LED strips (12 V) were sourced from a commercial retailer and around 3 strips were stuck into the inside of a 15 cm diameter copper ring. This was then placed on top of a magnetic stirrer with a small gap to allow a vent for cooling which was supplied by a CPU fan. Vials were placed ~1 cm from the LED strips.

3 DLS size, zeta potential and STEM sizes

Table S1: DLS and zeta potential measurements of FLPDA samples. Errors are the standard deviation of the triplicate data. Sizes from STEM images were determined from the mean of >100 measurements of spherical particles, with the associated error being the standard deviation.

Sample	Monomer molar ratio (DA:FLDA)	Hydrodynamic size / nm	PDI	Zeta potential / mV	Size (STEM) / nm
FLPDA	5:1	495.5 ± 23.05	0.382	-37.7 ± 0.8	207 ± 53
PDA	1:0	155.0 ± 1.12	0.044	-55.0 ± 1.2	110 ± 18

4 Fluorescence Calibration Curve

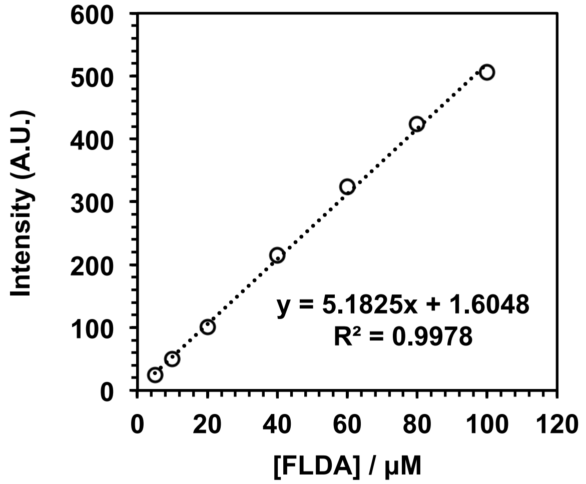

Figure S2: Fluorescence calibration curve of **FLDA** in water.

Table S2: Correlation data obtained from the calibration curve.

Intensity @ λ_{527} (0.1 mg/mL FLPDA)	Relative [FLDA] (µM)	Approximate FLDA content (nmol/mg FLPDA)*
146.58	27.97	279.7

**All measurements were carried out in water and 200 µL total volume.*

Control experiments

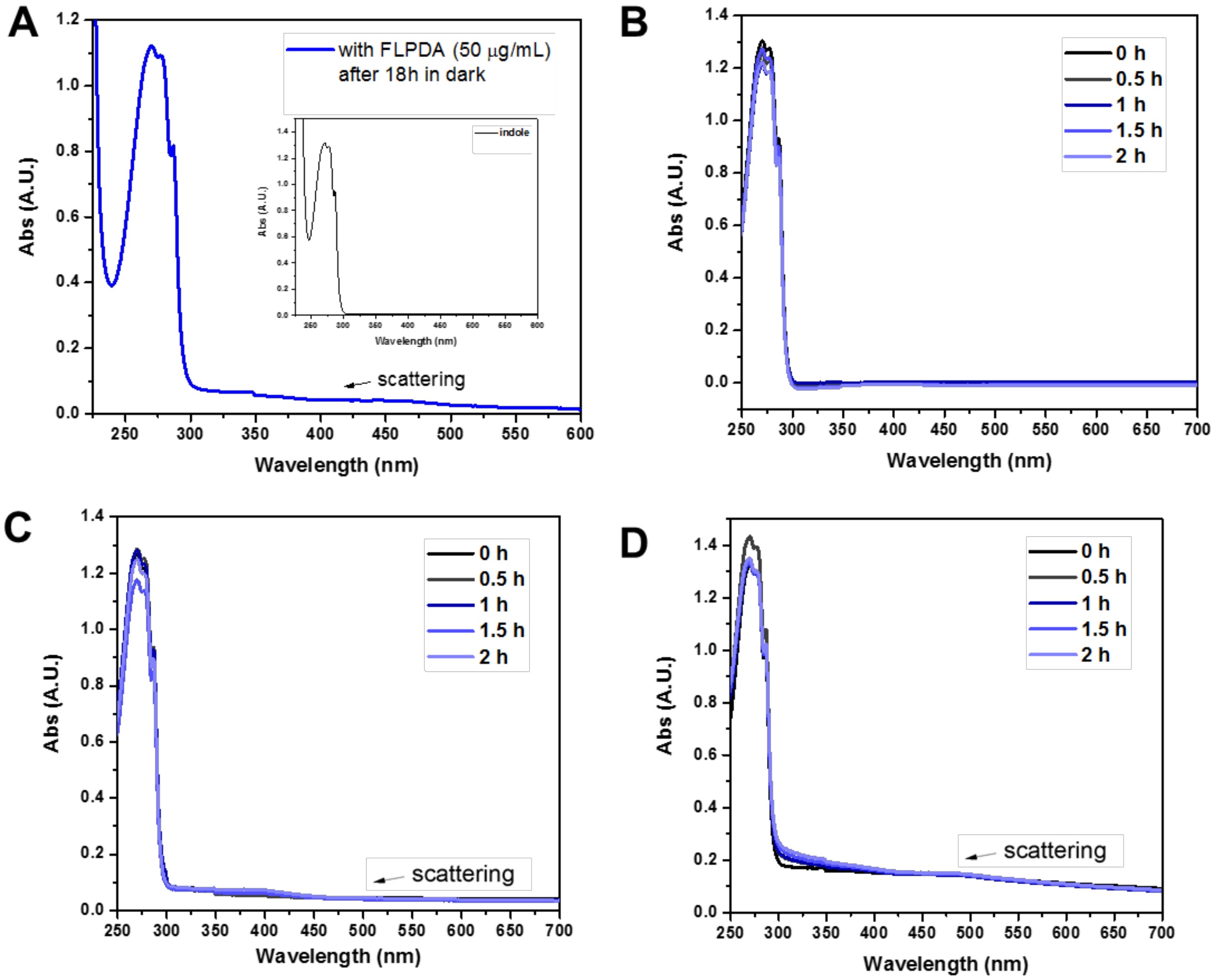

Figure S3: (A) 1.0 mM indole, 50 µg/mL FLPDA, ACN/H_2O (1:1, v/v, 2 mL), O_2 sat., RT, Dark, 10x Dilution; (B) 1.0 mM indole, 50 µg/mL FLPDA, ACN/H_2O (1:1, v/v, 2 mL), O_2 sat., RT, Blue LED Strips (12 V), 10x dilutions; (C) 1.0 mM indole, 50 µg/mL FLPDA, ACN/H_2O (1:1, v/v, 2 mL), Ar sat., RT, Blue LED Strips (12 V), 10x dilutions; (D) 1.0 mM indole, 50 µg/mL PDA, ACN/H_2O (1:1, v/v, 2 mL), O_2 sat., RT, Blue LED Strips (12 V), 10x dilutions.

H_2O_2 assay was carried out by first creating a standard calibration curve using Amplex Red (Ampliflu Red, Sigma-Aldrich, 100 µM), HRP (0.25 µM) and H_2O_2 (0-100 µM). Phosphate buffer (pH 7.4) was used to adjust final well volumes to 100 µL. A microplate reader (Tecan Spark) was used to record the fluorescence signals of resorufin (λ_{ex} = 540 nm, λ_{em} = 585 nm). To determine H_2O_2 amounts after irradiation, an aliquot (50 µL) of a reaction mixture was taken and used under the same conditions as above, comparing the fluorescent intensity of resorufin to the calibration curve.

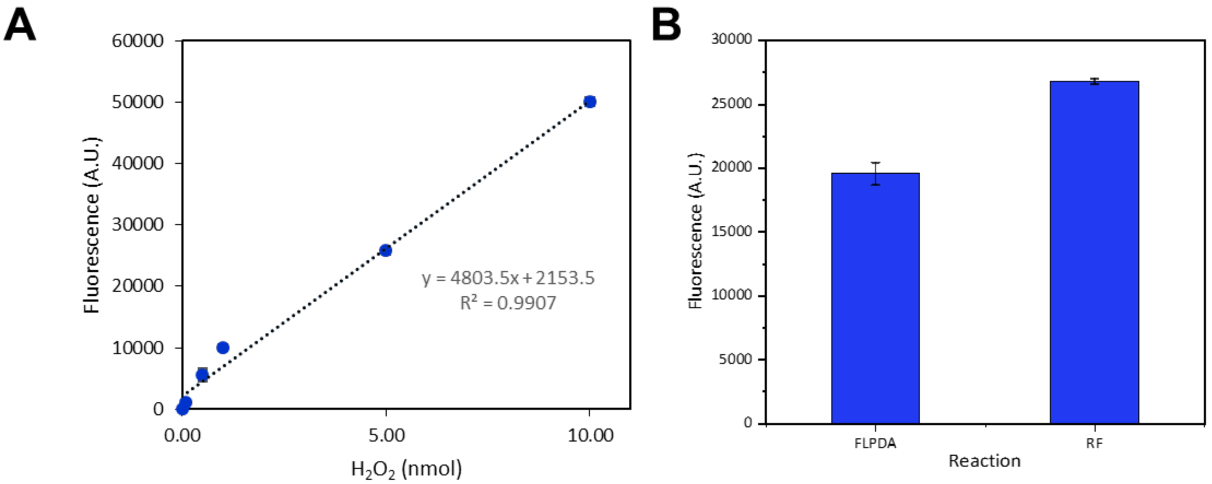

Figure S4: (A) H_2O_2 Calibration Curve (B) Fluorescence values for reactions post irradiation. All error bars are the standard deviation of triplicate data.

5 Indole adducts identified by LC-MS

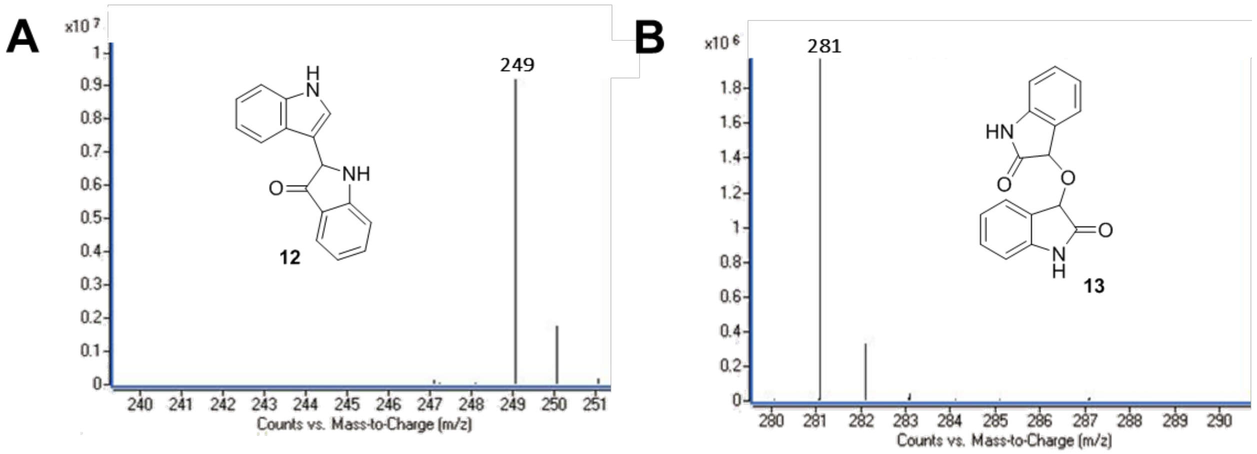

Figure S5: LC-MS chromatograms of (A) indole dimer **12** and (B) indole dimer **13.**

6 References

1 L. Deng, O. Norberg, S. Uppalapati, M. Yan and O. Ramström, *Org. Biomol. Chem.*, 2011, **9**, 3188–3198.

2 B. Geiseler and L. Fruk, *J. Mater. Chem.*, 2012, **22**, 735–741.

3 C. C. Le, M. K. Wismer, Z. C. Shi, R. Zhang, D. V. Conway, G. Li, P. Vachal, I. W. Davies and D. W. C. MacMillan, *ACS Cent. Sci.*, 2017, **3**, 647–653.
